# Digital Health Innovation and Climate Change: Designing Resilient Ecosystems with Digital Indicators

**DOI:** 10.1055/s-0046-1816590

**Published:** 2026-11-03

**Authors:** Dana Nehama, Gur Hanan, Nimrod Tachnai, Julien Grosjean, Stéfan J. Darmoni, Oscar Tamburis, Arriel Benis

**Affiliations:** 1 Faculty of Industrial Engineering and Technology Management, Holon Institute of TechnologyHolonIsrael; 1 Department of Data Science, Holon Institute of TechnologyHolonIsrael; 3 Department of Digital Health, Rouen University Hospital & AIMS Lab, Rouen UniversityNormandyFrance; 4 Institute of Biostructures and Bioimaging, National Research CouncilNaplesItaly; 5 Department of Digital Medical Technologies, Holon Institute of TechnologyHolonIsrael

**Keywords:** One Digital Health, One Health, Digital Health, Climate Change, Ecosystem

## Abstract

**Objective:**

This work aims to highlight the need to integrate One Digital Health (ODH) as a proactive response to the pressing health challenges of climate change. It emphasizes how digital indicators within the ODH framework can empower healthcare systems and ecosystem management decision-makers to become more resilient and adaptable while honoring the profound interconnectedness of human, animal, and environmental health from a digital technology viewpoint.

**Methods:**

A comprehensive literature review through digital technology demonstrated the deep interconnectedness of climate change’s impact on human, animal, and environmental health. We also showcased case studies where ODH principles and digital indicators have been successfully implemented to forge a path toward climate resilience and empower data-driven decision-making.

**Results:**

ODH is an essential framework for enhancing disease surveillance, monitoring the environment, and improving healthcare delivery through digital technologies. Case studies demonstrate how ODH can lead to better health outcomes, support ecosystem conservation, and facilitate more effective responses to climate-related challenges. Additionally, this research identifies digital indicators reflecting human, animal, and ecological health outcomes potentially related to climate change impacts. These ODH-driven indicators can have the potential to significantly enhance the responsiveness of global health systems and enable proactive interventions against health threats caused by climate change.

**Conclusion:**

Applying the ODH framework harnesses digital innovations to effectively address the complex health challenges intensified by climate change, thereby strengthening health and ecological systems and ensuring sustainable outcomes. Future research must focus on the global scaling of ODH interventions to enhance resilience across diverse healthcare settings. Furthermore, incorporating and standardizing digital indicators within the ODH framework will significantly strengthen health systems’ adaptability and proactively mitigate the health impacts of climate change.

## 1. One Digital Health as a Response to Climate Change

### 1.1 The Impact of Climate Change on Global Health

Climate change presents an escalating challenge to global health, exacerbating human, animal, and environmental vulnerabilities. Rising global temperatures and extreme weather events contribute to increased heat-related illnesses, respiratory diseases, proliferation of vector-borne infections, and mental health challenges [ 1 , 2 ]. The expansion of mosquito habitats due to warming climates has triggered dengue and malaria outbreaks in new regions, placing additional strain on public health systems [ 3 ]. Furthermore, climate-induced food and water insecurities heighten malnutrition rates, disproportionately affecting vulnerable populations such as children, older adults, and low-income communities [ 4 ]. These pressing health concerns underscore the necessity of climate-resilient healthcare systems that can adapt to evolving environmental threats.

Climate change significantly disrupts animal health by altering habitats, food availability, and disease transmission patterns [ 5 , 6 ]. Rising temperatures and prolonged droughts increase heat stress in livestock, reducing productivity and threatening food security [ 7 , 8 ]. Wildlife populations face habitat fragmentation and shifting migration patterns, increasing their exposure to novel pathogens [ 9 ]. These disruptions threaten biodiversity and elevate the risk of zoonotic disease spillover, reinforcing the intricate link between human, animal, and environmental health.

The degradation of ecosystems driven by climate change weakens biodiversity and essential environmental services, such as water purification and carbon sequestration. [ 10 , 11 ] Nevertheless, it is critical to remember that the global healthcare system is a significant emitter of greenhouse gas emissions (GHGs), with national averages worldwide accounting for 5% of Carbon dioxide (CO2) [ 1 , 12 , 13 ]. Rising sea levels, ocean acidification, and deforestation further destabilize ecosystems, endangering global food supplies and livelihoods. Without proactive intervention, these environmental challenges will continue to escalate, exacerbating the health burdens communities worldwide face. [ 14 ]

Given the profound health implications of climate change, there is an urgent need for innovative and integrated approaches to mitigate risks and enhance resilience. This is where ODH emerges as a transformative framework. By harnessing digital technologies for integrated monitoring, predictive modeling, and adaptive interventions, ODH offers a structured framework to address climate-related health challenges. The following section delves into how ODH can operationalize these strategies, fostering a sustainable and data-driven approach to safeguarding global health [ 15 ].

### 1.2 One Digital Health Helps to Tackle Climate Change’s Impact on Healthcare

One Health is a collaborative, multi-sectoral approach that recognizes the interconnectedness of human, animal, and environmental health. It emphasizes cooperation in public health, veterinary, and environmental science to address health threats like zoonotic diseases and antimicrobial resistance [ 16 ]. In parallel, Digital Health harnesses modern technologies such as mobile apps, telemedicine, artificial intelligence, and electronic health records to enhance healthcare delivery, improve patient outcomes, and support data-driven decision-making [ 17 , 18 ]. Bringing these two concepts together, One Digital Health (ODH) integrates the principles of One Health with Digital Health innovations, creating a unified framework that strengthens disease surveillance, environmental monitoring, and healthcare accessibility while promoting sustainable, interconnected health solutions across human, animal, and ecological systems [ 15 ].

The intersection of ODH and climate change represents a critical global health and sustainability frontier. By leveraging digital technologies, we can enhance our understanding of complex health-environment interactions and develop adaptive strategies to mitigate climate-related risks. As climate change accelerates, embracing the ODH framework is essential for building resilient ecosystems and safeguarding the health of future generations. Stakeholders across the healthcare and wellness sectors must collaborate to operationalize this framework and ensure a healthier, more sustainable future by developing literacy and practice of green and responsible medical informatics and digital health [ 19 ].

This collaboration allows both communities to benefit from efficient interactions via digital technologies, using near real-time data and decision support systems, aiming to support interventions and develop or improve policies. ODH looks at how a population (animal or human) impacts and interacts with an ecosystem and how the ecosystem responds accordingly. Advancements in mobile and universal tracking, reporting, and follow-up apps, web-based systems, and wearable devices have allowed citizens to engage and collaborate with governmental, health, environmental, and ecological organizations. Self-reporting platforms (e.g., social networks, health apps, environmental violations reporting apps) encourage citizens to report and share their data and allow learning opportunities so citizens can protect and take care of their health and well-being, as demonstrated in the COVID-19 outbreak, which has increased the awareness of the links and interactions between human health, animal health, and environmental health. This approach may help public health decision-makers understand how to develop efficient policies based on real-world and near–real-time data. Nowadays, household surveys, a longstanding data source, are embracing modern technologies and inclusive approaches, such as using telephone and web data collection methods to make them more efficient. Engaging respondents as co-creators empowers them to contribute to the data collection process [ 20 ].

### 1.3 One Digital Health as a Solution Framework

This paper explores the transformative potential of integrating digital indicators of human, animal, and ecological health in the face of escalating climate-related health challenges. By establishing the connections between ODH and health indicators based on digital technology advances, it becomes possible to highlight the potential of a proactive approach to improving public and environmental health in an era of climate uncertainty.

#### Integrated Monitoring and Surveillance

Digital technologies, such as remote sensing, wearables, and IoT sensors, act as catalysts within the technology ring of the ODH steering wheel, enabling comprehensive, real-time tracking of environmental changes and their implications for human, animal, and ecosystem health [ 15 ]. From an individual perspective (Individual perspective), wearables monitor physiological responses to climate stressors, such as heatwaves or air pollution, supporting early health interventions [ 21 ]. At the population level (Population perspective), satellite and sensor data facilitate the detection of deforestation, biodiversity loss, and zoonotic disease transmission risks. Ecosystem-wide integration of cross-domain data (Ecosystem perspective) enables proactive governance, ensuring timely policy responses and resource allocation to mitigate health and ecological threats [ 22 – 24 ].

#### Predictive Modeling and Data Integration

Machine learning and big data analytics, integral to the technology ring of ODH, enhance predictive capabilities for health risks associated with climate change, biodiversity shifts, and emerging infectious diseases [ 22 , 25 ]. AI-driven models analyze complex interactions among human, animal, and environmental health, identifying patterns that inform targeted interventions at individual and systemic levels (Human and Veterinary Healthcare, and Environment dimensions). For instance, predictive models have successfully forecasted malaria outbreaks using climate variables, enabling preventive measures at the population level. Digital dashboards and decision-support systems reinforce citizen engagement and governance (Citizen Engagement and Education dimensions) and improve healthcare policymaking and resource distribution by optimizing healthcare resource distribution and policy responses, ensuring resilience against climate and health crises [ 26 – 28 ].

#### Adaptive Interventions

Telemedicine, mobile health applications, and digital platforms are key components of digital healthcare transformation 4.0 (Industry 4.0 dimension), which enhances healthcare accessibility and adaptability in climate-affected and remote areas. At the individual level, mobile apps provide real-time health guidance, mental health support, and emergency response tools for displaced populations. For communities (Education and Citizen Engagement dimensions), digital education campaigns promote climate-adaptive behaviors, such as vector-borne disease prevention and sustainable agricultural practices, ensuring a well-informed population actively contributing to ODH. Systemically, these kinds of interventions improve health equity and resilience, aligning governance frameworks with emerging digital health solutions to strengthen overall adaptive capacity in the face of environmental and public health challenges [ 29 – 33 ].

## 2. The Challenge of Climate Change for Specific Ecosystems

By 2050, two-thirds of the world’s population will live in cities [ 34 ]. This trend requires more ground to build cities, causing natural area size decreases, increased air, water, and land pollution, and a collapse in wild animal populations, both related to a dramatic increase in natural resource consumption [ 35 ]. Despite these alarming observations, it has been consistently demonstrated that natural experiences are associated with a wide range of positive health outcomes. Urban nature can improve physical and mental health [ 36 ]. Previous studies show a positive link between living in cities near green spaces—such as parks, gardens, and nature reserves—and improved health indicators and reduced morbidity and mortality risks [ 37 , 38 ]. Thus, it seems that living close to green (e.g., community woodlands, green roofs, landscape around buildings, street trees, urban parks and gardens) and blue (e.g., urban water streams such as rivers, lakes, and coastal waters) spaces in urban areas, also contributes to the population who lives near them, as they also provide ecosystem services [ 39 , 40 ].

An ecosystem is a dynamic complex of plants, microorganisms, animals, humans, and non-living elements of the environment interacting as a functional unit. Urbanization affects ecosystems by minimizing nature, increasing building areas, polluting clean water resources, and more. As cities and populations grow, the demand for and consumption of biological and physical resources increases, and the impacts on ecosystems and their services escalate [ 41 – 43 ].

Ecosystem services refer to the benefits people obtain from ecosystems [ 41 ]. One example of these services relates to the ones that urban water streams (i.e., blue spaces) provide for the nearby population and environment. These services include provisioning services—providing habitats, water, and food supply to aquatic communities, birds, mammals, and plants. Moreover, green spaces are complementary and provide similar services. However, both blue and green spaces offer health-related services when we consider their positive effects on the mental health of inhabitants and visitors [ 44 , 45 ]. Furthermore, blue spaces such as urban water streams provide regulation (as a service) supporting climate change mitigation, such as reducing heat, filtering the air, and participating in flood mitigation by effectively draining the surface runoff of rainwater during extreme precipitation events. Additionally, ecosystem services are culturally related, which refers to nonmaterial benefits, including physical and mental benefits for the population in their neighborhoods [ 46 – 48 ]. In that term, green and blue spaces in urban environments provide mental and physical benefits as they offer a place for educational activities, social interactions, and areas to practice sports and leisure activities; they also provide therapeutic experiences—serving as havens for stress reduction, improving health and well-being while interacting and connecting with nature, the environment, and animals [ 49 ]. These nonmaterial benefits can substantially affect humans’ well-being at different scales, manifesting differently [ 32 , 43 ].

### 2.1 Urban ecosystems: OneAquaHealth

OneAquaHealth (OAH) is an EU-funded project that aims to improve the sustainability and integrity of freshwater ecosystems in urban environments by investigating the interconnectedness of ecosystem health and human well-being [ 50 ]. Essentially, OAH focuses on understanding how the health of urban streams and rivers impacts human health and how to improve both [ 50 ]. ODH plays a pivotal role in this project. ODH offers a comprehensive approach that leverages cutting-edge digital tools to monitor, analyze, and improve the health of freshwater ecosystems in urban environments. Thus, ODH gives the theoretical framework to guide the data collection and management related to ecological systems (i.e., environmental pollutants, water quality, biodiversity) and human activity. As a critical component of OAH, data is instrumental in identifying risks and pinpointing areas that require intervention.

The contributions directly related to ODH employ advanced AI (Artificial Intelligence) techniques and data analytics, such as machine learning [ 51 , 52 ], Large Languages Models (LLMs) [ 53 – 55 ], and Geographic Information Systems [ 56 , 57 ] to analyze some of the collected data. This enables human health, ecological, and policy-making researchers to uncover hidden patterns and better understand environmental changes, leading to the early warning signs identification of ecosystem distress. Furthermore, ODH looks globally and specifically as a part of the OAH project to the FAIR principles for enhancing the data exchanges and facilitating the translation of complex information into clear and understandable formats [ 58 – 60 ]. This empowers decision-makers and the public to grasp the current state of freshwater ecosystems and take appropriate action [ 49 , 61 ]. By integrating these elements, the ODH framework fosters a collaborative environment in OAH where researchers, policymakers, and citizens work together to safeguard freshwater ecosystems and public health. OneAquaHealth exemplifies the power of ODH in action.

In conclusion, ODH is a cornerstone for OneAquaHealth and climate change initiatives. Medical informatics and AI are the backbone of ODH, providing the analytical muscle to transform raw data into actionable insights. This empowers the protection of freshwater ecosystems, ensuring a healthier planet for all.

### 2.2 Impact of Heat Waves on Diabetes: from Observation to Analysis of Pathological Podels

As part of the Rouen-Normandie University’s (URN) commitment to ecological transition for sustainable development, the “TRANSITION” project, funded through the France 2030 program “Excellence in All Its Forms,” aims to develop innovative and interdisciplinary research to address multi-risk challenges in the context of environmental change. This initiative supports evidence-based decision-making for both local and national actors. The DIAB-HEAT project aims to accelerate interdisciplinary research within this dynamic, reinforcing collaborations among URN research units on risk assessment and territorial adaptation [ 62 ].

In 2019, heat-related mortality rose by over 80% among individuals over 65 years old. The Intergovernmental Panel on Climate Change projects a sharp increase in extreme heat days by the end of the century, exacerbating risks for populations with chronic diseases like type 2 diabetes [ 1 , 2 ]. High temperatures can disrupt glucose homeostasis by impairing thermoregulation, increasing dehydration, and altering insulin sensitivity [ 63 ]. Furthermore, the hypothalamic neuroendocrine systems, which play a key role in blood sugar regulation, may be particularly vulnerable to extreme heat [ 64 , 65 ]. Addressing this emerging health risk requires integrating climate resilience with digital health solutions, particularly in the context of smart cities and advanced healthcare systems. The increasing frequency and intensity of heat waves underscore the need for smart healthcare systems that leverage the Internet of Medical Things (IoMT), remote monitoring, and telemedicine to protect at-risk populations [ 64 , 66 – 69 ]. Access to Electronic Health Records (EHRs) and Patient-Reported Outcome Measures (PROMs) can enable real-time tracking of diabetes management and early detection of heat-related complications [ 70 , 71 ]. Smart cities with environmental sensors and AI-driven analytics can provide personalized heat advisories and adaptive interventions for vulnerable individuals [ 32 , 52 , 72 ].

The DIAB-HEAT project seeks to examine the effects of heat waves on the pathophysiology of diabetes using a multidisciplinary approach. The initiative will start with an epidemiological analysis of extreme heat events and their correlation with diabetes incidence across local, regional, and national levels. This analysis will consider various meteorological factors, including temperature, humidity, and the duration of heat waves, as well as air quality indicators. To deepen our understanding, experimental studies will be undertaken using healthy and diabetic murine models to assess their neuroanatomical and physiological responses to heat stress. These experimental findings will be compared to real-world meteorological data to ensure a comprehensive understanding of the disease mechanisms involved.

Finally, the project will investigate the impact of actual heat wave episodes on diabetic patients, further validating findings under controlled conditions using obese and diabetic animal models. This systematic approach will provide invaluable insights into how extreme heat influences diabetes management and outcomes. This research will enhance the development of personalized prevention strategies and real-time interventions by integrating digital health solutions such as telemedicine and wearable biosensors. Advanced healthcare infrastructures, including remote monitoring platforms and AI-driven predictive modeling, are crucial for addressing the health impacts of climate change. Fashioning synergy between climate adaptation, IoMT, and precision medicine will facilitate a proactive approach to managing diabetes in the face of extreme environmental conditions. This innovative framework aims to improve patient outcomes and ensure effective responses to the challenges posed by climate change.

## 3. Digital Technologies as One Health Indicator

### 3.1 Human Health Digital Indicators

Interest in measuring population health has a long history. Hippocrates noted environmental factors affecting endemic diseases, while the English Sanitary Act of 1388 aimed to control parasites. Early quarantines in Marseilles, France, in 1377 and Venice, Italy, in 1403 highlighted the need for population-level health interventions. In 17th century London, clerks created weekly reports on burials and death causes, taking action during plague outbreaks based on this data. Birth and death records also helped governors with annuities, electoral boundaries, taxation, and inheritance issues. National surveys began in the 1950s, focusing on population health indicators to inform disease control policies [ 73 ].

Health data is essential for creating population health indicators vital for public health decision-making. These indicators help national health authorities set goals and provide crucial information on health risks and trends to governments, healthcare professionals, and the public. The goal is to improve population health through advocacy, accountability, quality management, and research [ 73 ]. Health indicators include direct measures like physical activity and behavior, diseases, and mortality at the individual and population levels, as well as indirect measures like technological engagement and socio-economic changes. In developed countries, the focus is on lifestyles and behaviors, while in developing countries, it is on illness and mortality rates [ 74 ]. Nowadays, looking at health indicators from a digital perspective and considering “health digital indicators” (HDIs) is crucial. Therefore, a large part of the health indicators is available through clinical information systems and electronic health records, and a large part of the data is collected through digital health tools, comprising mobile and ubiquitous health (mHealth and uHealth) applications, wearable devices, and other Internet of Things (IoT) technologies. Digital health solutions facilitate (near-) real-time patient monitoring, broadly increase healthcare service customers’ engagement, and enhance healthcare accessibility. [ 75 – 77 ] The Patient Reported Outcome Measurements (PROMs) surveys are another way to get digital health measurements. [ 78 ] Thus, this reporting method of patients’ experiences is essential, as it is seen as an important mechanism for holding healthcare providers accountable for their performance and empowering patients to act as wise consumers [ 79 , 80 ].

From the viewpoint of exploring the connection between ODH and Climate Change, it is essential to consider that with the increasing integration of digital technologies in healthcare, HDIs provide insights into personal and population health as well as allow taking into account environmental influencing factors (e.g., location, ecological neighborhood, human/animal interactions). Thus, below, we list a set of exemplary HDIs - in other words, health indicators generated or collected by using digital technologies according to the ODH perspectives and by considering climate change impacts ( Table 1 ).

**Table 1:** Number of papers collected in the PubMed database from 2013 to 2023 for each country based on the first author’s affiliation country.

**ODH Perspective**	**ODH** **Dimension(s)**	**Indicator**	**Definition from the climate change standpoint**
Individual Health and Wellness	Citizen Engagement and Education	Percentage of patients having or using an electronic health record (EHR) or a personal health record (PHR)	Engagement in self-health management via digital patient portals indicates a need for EHR and PHR. Understanding how climate change impacts personal healthcare needs may be possible.
		Digital health literacy levels	Measures individuals' ability to navigate digital health tools, including understanding climate-related health risks.
		Social network engagement in health topics (posts, likes, shares, comments)	Assesses citizen participation in health discussions, awareness campaigns, and digital health advocacy, particularly regarding climate-related health challenges.
	Human Healthcare: Digital Biometrics	Heart rate variability (HRV)	Monitors stress, cardiovascular health, and fitness through wearable devices (e.g., smartwatches and fitness trackers), considering climate-related stressors.
		Step count and physical activity levels	Tracks mobility and sedentary behavior using accelerometers and fitness apps, highlighting the impact of climate conditions on physical activity.
		Sleep patterns and quality	Evaluates sleep disturbances through wearable sensors and smart mattresses, factoring in climate-related sleep disruptions such as heat waves.
		Blood oxygen levels (SpO2)	Helps detect respiratory and cardiovascular conditions via pulse oximeters integrated into smart devices, especially in response to air pollution and climate change.
		Continuous glucose monitoring (CGM) data	Essential for diabetes management using CGM sensors and smart insulin pumps, considering climate impacts on food security and metabolic health.
		Time spent on health-related apps and self-monitoring tools	Measures user engagement with mobile applications for fitness tracking, nutrition logging, and symptom monitoring relevant to climate-driven health adaptations.
	Human Healthcare: Digital Mental Health	Use of mental health apps	Tracks engagement with digital therapy tools and cognitive behavioral therapy (CBT) applications, addressing climate anxiety and eco-stress.
		Social media sentiment analysis	Uses AI to assess linguistic patterns indicating mental well-being, including responses to climate-related stressors.
		Screen time and digital addiction metrics	Evaluates excessive digital consumption's impact on mental health through smartphone tracking apps, considering increased online engagement due to climate disasters.
		Time spent on social media and its psychological impact	Measures the influence of prolonged social media use on mental well-being, stress levels, and self-perception, particularly concerning climate crisis discussions.
Population and Society	Human Healthcare, Healthcare Industry 4.0, Education: Telehealth and Remote Monitoring	Frequency of telemedicine consultations	Measures digital healthcare accessibility via video conferencing and remote health platforms, considering climate-related barriers to in-person care.
		Patient adherence to remote monitoring	Assesses compliance with digital health programs, such as remote blood pressure monitoring and wearable ECG devices, to address climate-induced health concerns.
		Usage of AI-powered diagnostics	Evaluates AI-driven clinical decision support systems in disease diagnosis and treatment planning, incorporating climate-related disease patterns.
	Healthcare Industry 4.0, Education, Citizen’s Engagement: Health Data Sharing and Interoperability	Interoperability of EHRs	Measures seamless data exchange between providers using HL7 FHIR standard, ensuring integration of climate-related health data.
		Patient trust and privacy concerns	Assesses confidence in digital health security through surveys, particularly regarding climate-related health data sharing.
	Healthcare Industry 4.0, Education, Citizen’s Engagement: Digital Health Equity and Accessibility	Access to mobile health applications by demographics	Identifies gaps in digital healthcare accessibility, particularly for communities disproportionately affected by climate change.
		Geographic distribution of telehealth adoption	Highlights disparities in rural vs. urban digital healthcare use, factoring in climate-driven migration and infrastructure challenges.
Ecosystem	Healthcare Industry 4.0, Environment: Environmental Factors and Health Technology Integration	Use of AI chatbots for initial diagnosis	Monitors AI's role in preliminary patient assessments via virtual assistants, with applications in climate-related health risk assessments.
		Effectiveness of AI-generated health recommendations	Evaluates the accuracy of AI-driven medical insights, ensuring responsiveness to climate-related health conditions.

### 3.2 Animal Health Indicators

The health of both wild and domestic animals is essential in the context of One Health and the prevention of zoonotic diseases. Digital indicators are vital tools for monitoring, predicting, and mitigating disease outbreaks in animal populations, facilitating early intervention, and lowering the risk of transmission to humans. One recent approach involves wearable biosensors that track vital signs in livestock, such as heart rate, respiratory rate, and body temperature, which enable early disease detection [ 81 , 82 ]. Additionally, AI-driven sound detection systems can analyze animal vocalization patterns, helping identify respiratory issues before visible symptoms appear [ 83 , 84 ]. Thermal imaging cameras detect subtle temperature variations indicating potential infections or inflammation [ 85 ]. On the practical side, mobile health apps for livestock management provide real-time alerts for necessary veterinary interventions, helping to diminish the likelihood of disease outbreaks on farms. Furthermore, automated diagnostic platforms utilize AI technology to quickly detect animal parasites and bacterial infections, thus expediting treatment and minimizing transmission risks [ 86 ].

Digital technologies are revolutionizing One Health by enhancing our ability to predict, monitor, and respond to zoonotic threats, biodiversity loss, and climate change-related health risks. By integrating AI, IoT, and real-time analytics, we can develop more resilient ecosystems that safeguard human, animal, and environmental health for future generations [ 87 ]. Moreover, discussing animal health digital indicators means considering the monitoring of zoonotic diseases to surveil the risk of transmission from animals to humans or reciprocally. This is related to significant threats to human health, such as vector-borne diseases. Emerging infectious diseases in wildlife, domestic animals, plants, or people can be intensified by human activities such as land clearing and habitat fragmentation or the overuse of antibiotics, driving the rapid evolution of antibiotic resistance in many bacterial pathogens. The deterioration of nature and consequent disruption of benefits to people has both direct and indirect implications for public health ( Table 2 ) [ 88 ].

**Table 2:** Exemplary Animal Health Digital Indicators from the ODH Perspectives and Dimensions.

**Dimension**	**Indicator**	**Definition from the climate change standpoint**
Citizen Engagement	Number of Users in Digital Environmental Citizen Science Platforms	Measures public participation in ecological monitoring through mobile apps and online platforms, fostering awareness of climate-related health risks affecting humans and animals.
	Social Media Mentions and Sentiment Analysis on Climate and Biodiversity	Tracks engagement levels, awareness campaigns, and advocacy efforts related to ecological health, linking environmental degradation to emerging health threats.
Education	Percentage of Population with Digital Literacy in Environmental Health Data	Assesses individuals' ability to interpret and use digital ecological data effectively, promoting informed decision-making to mitigate climate-related health impacts on human and animal populations.
Environment	IoT Sensor-Based Water Quality Index (WQI) Monitoring Rate	Tracks pH, temperature, dissolved oxygen, and pollutants in freshwater ecosystems using IoT sensors, assessing risks of contaminated waterborne diseases for human and animal health.
	AI-Powered Air Quality Index (AQI) Alerts Generated Per Day	Measures air pollution levels (e.g., PM2.5, CO2, NO2) and their impact on ecological, human, and animal respiratory health, particularly in areas affected by climate change.
	Number of Remote Sensing Reports on Biodiversity Loss Per Year	Uses satellite imagery and drones to monitor species distribution and habitat changes, evaluating biodiversity loss impacts on ecosystem resilience and zoonotic disease transmission.
	AI-Based Harmful Algal Bloom Monitoring Alerts Per Month	Uses machine learning and satellite data to detect toxic algal blooms, reducing risks to aquatic ecosystems, drinking water supplies, and seafood safety for human and animal health.
(Human and) Veterinary Healthcare	Digital Surveillance Reports on Environmental Pollution and Zoonotic Disease Outbreaks Per Year	Analyzes correlations between ecosystem degradation and the spread of zoonotic pathogens using digital tracking, aiding in early detection and prevention of outbreaks linked to climate change.
	Number of Remote Sensing Detections of Wildlife Health Anomalies Per Month	Uses thermal imaging and satellite data to detect health changes in animal populations, serving as an early warning system for climate-driven disease spillovers to humans.
Healthcare Industry 4.0	AI-Powered Extreme Weather Event Predictions Per Year	Uses machine learning to forecast hurricanes, droughts, and floods based on climate trends, enabling proactive responses to prevent health crises in both human and animal populations.
	Number of Digital Reports on Environmental Issues via Mobile Apps	Utilizes mobile apps for real-time reporting of pollution, illegal deforestation, and biodiversity loss, helping mitigate environmental factors contributing to vector-borne diseases.
	Cloud-Based Interoperability Score of Ecological Databases	Ensures seamless integration and sharing of environmental data across scientific and policy platforms, enhancing the capacity for cross-sectoral responses to climate-related health challenges.

### 3.3 Ecological Health Digital Indicators

Ecosystems provide ecological goods and services for human health, such as air purification and freshwater supply. Ecological indicators measure an ecosystem’s state (or health) and are primarily used to characterize status and track or predict significant changes and trends. Ecological indicators reflect chemical, physical, biological, and globally ecosystem attributes. For example, considering water sources, chemical and physical indicators measure components such as water pH, water clarity and salinity, temperature, suspended sediments, dissolved oxygen, eco-toxicants, and nutrients. In contrast, biological indicators, also known as bioindicators, refer to organisms, species, or communities, like harmful algal blooms, macroinvertebrates, aquatic invasive species, and fish types and their abundance. As in human health, indicators are critical tools for assessing the health of ecological systems, identifying specific problems, and driving management actions and interventions [ 89 , 90 ].

Ecological health evaluation typically involves examining species diversity, ecological functions and processes, biogeochemical cycles, ecosystem services and resources, and ecosystem resilience and stability [ 91 ]. Today, digital tools allow ecosystem data collection in different ways, such as sensor networks [ 92 – 94 ] or remote sensing satellites to monitor flooding, sea ice, wave heights, and ocean temperature, as well as satellite imagery and drones for mapping wetlands and monitoring land use [ 95 ].

As a part of the Citizen science approach, additional tools such as mobile applications are used for tasks consisting of collecting data relating to the natural world by the grand public as active partners of scientific projects and actions. Apps and platforms used through mobile devices enable citizens to provide timely geospatial information that contributes to scientific understanding and decision-making for environmental and biodiversity citizen science. [ 96 ]

As a part of the digital landscape, ecosystem health analysis applied data science plays a critical part [ 97 ]. For example, the GLOBE Data Entry app, developed by NASA, lets users record measurements in the field using their phone’s camera and GPS. This framework helps verify satellite data and encourages citizen engagement with environmental health. Additionally, there are apps for reporting environmental issues [ 98 ]. Future health ecosystems require the timely introduction of digital tools to investigate complex biological systems. Emphasizing education for healthcare professionals, computer scientists, and decision-makers will enhance data collaboration and analysis. This approach aims to interconnect animal, human, and environmental health, harnessing real-time data collection to improve early infection detection in areas like agriculture and conservation biology [ 99 ].

Table 3 reports a set of exemplary ecological digital indicators generated or collected by using digital technologies for the ODH dimensions and by considering climate change impacts.

**Table 3:** Exemplary Ecological Digital Indicators from the ODH Perspectives and Dimensions.

**ODH Dimension**	**Indicator**	**Definition from the climate change standpoint**
Citizen Engagement	Number of Users in Digital Environmental Citizen Science Platforms	Measures public participation in ecological monitoring through mobile apps and online platforms, fostering awareness of climate-related health risks affecting humans and animals.
	Social Media Mentions (posts, likes, shares, comments) and Sentiment Analysis on Climate and Biodiversity	Tracks engagement levels, awareness campaigns, and advocacy efforts related to ecological health, linking environmental degradation to emerging health threats.
Education	Percentage of Population with Digital Literacy in Environmental Health Data	Assesses individuals' ability to interpret and use digital ecological data effectively, promoting informed decision-making to mitigate climate-related health impacts on human and animal populations.
Environmental Factors	IoT Sensor-Based Water Quality Index (WQI) Monitoring Rate	Tracks pH, temperature, dissolved oxygen, and pollutants in freshwater ecosystems using IoT sensors, assessing risks of contaminated waterborne diseases for human and animal health.
	AI-Powered Air Quality Index (AQI) Alerts Generated Per Day	Measures air pollution levels (e.g., PM2.5, CO2, NO2) and their impact on ecological, human, and animal respiratory health, particularly in areas affected by climate change.
	Number of Remote Sensing Reports on Biodiversity Loss Per Year	Uses satellite imagery and drones to monitor species distribution and habitat changes, evaluating biodiversity loss impacts on ecosystem resilience and zoonotic disease transmission.
	AI-Based Harmful Algal Bloom Monitoring Alerts Per Month	Uses machine learning and satellite data to detect toxic algal blooms, reducing risks to aquatic ecosystems, drinking water supplies, and seafood safety for human and animal health.
Human & Veterinary Healthcare	Digital Surveillance Reports on Environmental Pollution and Zoonotic Disease Outbreaks Per Year	Analyzes correlations between ecosystem degradation and the spread of zoonotic pathogens using digital tracking, aiding in early detection and prevention of outbreaks linked to climate change.
	Number of Remote Sensing Detections of Wildlife Health Anomalies Per Month	Uses thermal imaging and satellite data to detect health changes in animal populations, serving as an early warning system for climate-driven disease spillovers to humans.
Industry 4.0 Technologies	AI-Powered Extreme Weather Event Predictions Per Year	Uses machine learning to forecast hurricanes, droughts, and floods based on climate trends, enabling proactive responses to prevent health crises in both human and animal populations.
	Number of Digital Reports on Environmental Issues via Mobile Apps	Utilizes mobile apps for real-time reporting of pollution, illegal deforestation, and biodiversity loss, helping mitigate environmental factors contributing to vector-borne diseases.
	Cloud-Based Interoperability Score of Ecological Databases	Ensures seamless integration and sharing of environmental data across scientific and policy platforms, enhancing the capacity for cross-sectoral responses to climate-related health challenges.

## 4. Discussion

The Sustainable Development Goals (SDGs) provide a global framework for addressing critical challenges, including health, climate change, and technological advancements [ 100 ] . SDG indicators are measurable benchmarks to track progress toward these goals, yet their relevance varies across regions. In advanced countries, where healthcare and digital infrastructure are already well-developed, some SDG indicators may be of limited applicability [ 101 ]. Similarly, from an ODH point-of-view, which integrates digital innovations with One Health principles to tackle climate-driven health challenges, existing SDG indicators do not fully capture the complexity of digital and ecological health interactions [ 102 , 103 ]. The limitations of SDG indicators are also evident when considering global biodiversity targets. The Aichi Biodiversity Targets (2011–2020), [ 104 ]designed to halt biodiversity loss and promote sustainable ecosystems, aligned with several SDGs, particularly SDG 14 (Life Below Water) [ 105 ] and SDG 15 (Life on Land). [ 106 , 107 ] However, by 2020, none of these targets were fully met, revealing implementation, monitoring, and accountability gaps. In response, the Kunming-Montreal Global Biodiversity Framework (GBF) was introduced in 2022, outlining new commitments such as protecting 30% of terrestrial and marine areas by 2050, reducing pollution, and ensuring sustainable resource use. [ 108 ] While these targets align with broader SDG objectives, they lack robust digital health and ecological monitoring components, particularly in ODH. While technology-related SDG indicators, such as those on internet access, digital literacy, and ICT integration in healthcare, offer valuable insights [ 109 ], they often fail to address the nuanced role of digital health in ecosystem resilience and climate adaptation [ 110 ]. For example, biodiversity conservation efforts could greatly benefit from AI-powered surveillance systems, remote sensing technologies, and big data analytics to track species population trends, habitat degradation, and the spread of zoonotic diseases. As ODH projects leverage (near) real-time data, AI-driven predictive modeling, and digital surveillance to enhance health and environmental decision-making, developing more refined, interdisciplinary human, animal, and ecological indicators is crucial to reflect the transformative potential of emerging digital health technologies.

With its systemic and integrative approach, One Digital Health emphasizes the critical role of digital technologies in addressing climate-related challenges. The various monitoring systems and digital indicators above underline the importance of advanced technology-embedding in maintaining ecological balance and preventing climate change-related crises. Innovative approaches, such as wearable (bio)sensors for monitoring the effects of climate change on human, ecological, and animal health, use AI-driven tools and mobile applications that provide (near) real-time information in context to aid in managing individual, population, and ecosystem health and reducing or mitigating disruptive hazardous events [ 66 , 72 , 88 ].

Amid these challenges, numerous opportunities arise to advance ODH. Fostering interdisciplinary research and innovation is essential for enhancing data-driven decision-making. Integrating citizen science initiatives with digital platforms is expected to improve data collection, increase community engagement, and contribute to a more holistic understanding of climate change-related health trends.

One way to take these challenges to the next level and allow the indicators dissemination will consist of adding the different human, animal, ecological digital indicators health to the Medical Informatics and digital health Multilingual Ontology (MIMO) 1 a freely accessible tool designed to provide a multilingual controlled vocabulary specifically for the field of health informatics and digital health, aiming to improve international collaborations by facilitating communication across different languages within the medical domain [ 109 , 110 ]]. MIMO contains around 4,000 terms and concepts continuously updated and is translated as much as possible in more than 30 languages.

While digitalization, informatics, and AI-driven advancements, as One Digital Health supports offer transformative benefits, the environmental implications of AI infrastructure must not be overlooked. Developing and deploying computational systems and models require substantial computational power, leading to significant energy and water consumption. As AI infrastructure expands to meet increasing demand, its environmental footprint is expected to grow, intensifying the strain on global resources. Accordingly, to limit the negative impact of digitalization, informatics, and AI on human, animal, and ecosystem health, it is crucial to develop green and responsible digitalization, informatics, and AI to be eco-friendly as much as possible [ 19 , 111 ].

## 5. Conclusion

The overarching theme of this paper is the significance of integrating advanced digital technologies, including artificial intelligence and real-time analytics, to enable proactive responses to (human, animal, or ecosystem) health threats stemming from environmental changes. ODH advocates its digital-oriented holistic approach to safeguard public health, animal welfare, and ecosystem integrity in an increasingly unpredictable climate by fostering interdisciplinary collaboration and enhancing data sharing across sectors.

The intersection of One Digital Health and climate change represents a critical frontier in global health and sustainability. By leveraging digital technologies, we can enhance our understanding of complex health-environment interactions and develop adaptive strategies to mitigate climate-related risks. As climate change accelerates, embracing the One (Digital) Health framework is essential for building resilient ecosystems and safeguarding the health of future generations. Stakeholders across sectors must collaborate to operationalize this framework and ensure a healthier, more sustainable future.

## Footnotes

1
https://www.hetop.eu/hetop/rep/ko/EFMIMIMO/
